# The making and breaking of insect endogenous retroviruses

**DOI:** 10.1038/s44318-025-00472-7

**Published:** 2025-06-05

**Authors:** Rebecca Halbach, Ronald P van Rij

**Affiliations:** Department of Medical Microbiology, Radboud University Medical Center, Nijmegen, the Netherlands

**Keywords:** Evolution & Ecology, Microbiology, Virology & Host Pathogen Interaction, RNA Biology

## Abstract

A recent study uncovers how spatiotemporal expression and infectivity shape the evolution of endogenous retroviruses.

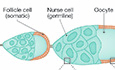

## Teaser

Retroelements are main constituents of eukaryotic genomes. Recent work by Senti et al (2025) reports that retrotransposons and endogenous retroviruses occupy specific spatiotemporal niches in the fly ovary, where they are kept in check by tissue-specific immune mechanisms. These findings suggest dynamic co-evolution of endogenous retroviruses and their hosts with relevance for their functional diversification.

A substantial fraction of eukaryotic genomes is comprised of transposable elements, especially retrotransposons, which replicate via an RNA intermediate in a copy-and-paste mechanism. Around one-third of the transposable elements in the fruit fly *Drosophila melanogaster* are potentially active (McCullers and Steiniger, 2017) and co-exist within the cellular ecosystem of an individual host. As genomic parasites that depend on vertical transmission, retrotransposons need to activate and transpose in the germline to ensure their evolutionary success. However, since transposon activity can lead to mutations, aberrant gene expression, genomic instability, and sterility, dedicated host defense mechanisms are in place to protect against this threat. In gonads, the PIWI-interacting (pi)RNA pathway is critical to control transposon activity (Sato and Siomi, 2020), and retroelement evolution is tightly linked to this host defense mechanism. The principles underlying transposon evolution and diversification are, however, not well understood.

**Figure 1:**
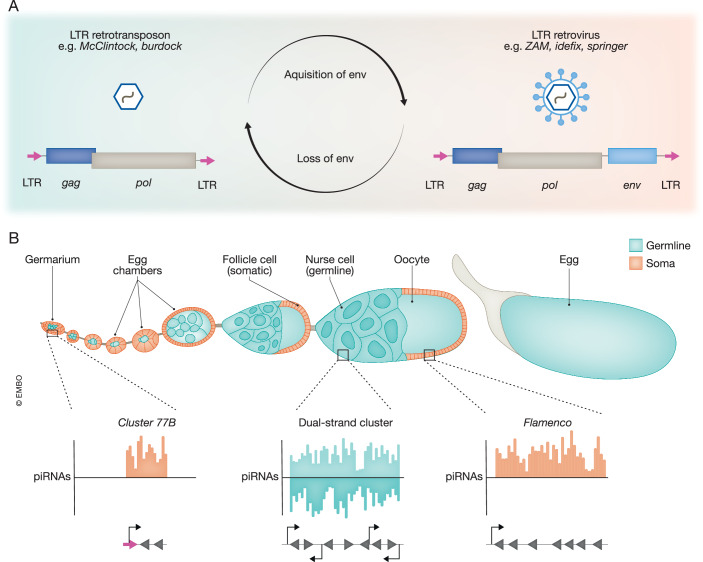
Evolution of endogenous retroviruses in the *Drosophila* ovary. (**A**) Genome structure of LTR retrotransposons (left) and endogenous retroviruses (right). The initial acquisition of an *envelope* (*env*) gene from an unrelated baculovirus led to the evolution of a clade of infectious endogenous retroviruses (*Errantivirus* genus or *gypsy/gypsy* clade), whereas the subsequent loss of the *env* gene resulted in non-infectious LTR retrotransposons. (**B**) Structure of the *Drosophila* ovary (top panel) and piRNA clusters expressed in different cell types. Endogenous retroviruses are expressed in the soma and targeted by piRNAs derived from the uni-strand piRNA clusters *flamenco* and *cluster 77B*, whereas LTR retrotransposons are active in the germline where dual-strand clusters are expressed.

A study by Senti et al (2025) now sheds light on the evolution of long terminal repeat (LTR) retrotransposons and endogenous retroviruses in *D. melanogaster*. The authors specifically focus on the clade of g*ypsy/Ty3* LTR retroelements, classified as *Metaviridae* by the International Committee on Taxonomy of Viruses. Within this family, members of the *Errantivirus* genus (also referred to as the *gypsy/gypsy* clade) are known to have acquired an additional trait: infectivity (Kim et al, 1994). Most *gypsy/Ty3* elements contain two genes: *gag*, encoding structural proteins required for capsid formation, and *pol*, which codes for proteins with protease, reverse transcriptase, and integrase activity. Yet, members of the *gypsy/gypsy* clade may encode a third gene: an *envelope* (*env*) gene that was acquired from an unrelated baculovirus. The envelope glycoprotein mediates cellular entry, and its acquisition transforms cell-autonomous retrotransposons into infectious endogenous retroviruses (Malik et al, 2000) (Fig. 1A). Through phylogenetic analyses and ancestral state reconstruction, the authors provide evidence that an *env*-containing endogenous retrovirus is the ancestor of the monophyletic *Errantivirus* genus, from which non-infectious retrotransposons independently evolved multiple times through loss of the *env* gene.

To analyze the spatiotemporal expression of individual *gypsy/gypsy* elements, the authors made use of flies in which the piRNA pathway was inactivated in specific compartments of the ovary. The *Drosophila* ovary is a complex organ containing the developing oocyte and the supporting nurse cells that transfer their cytoplasmic content to the oocyte (Fig. 1B). These two cell types constitute the fly germline, which is surrounded by follicle cells and other somatic cell types (Rust et al, 2020). These somatic cells form a niche for germline differentiation, establish egg polarity, and deposit the eggshell. Inactivation of the piRNA pathway allows retroelements to become active, after which they can be detected using single-molecule fluorescence in situ hybridization. Senti and colleagues (2025) found that expression of non-infectious retrotransposons lacking the *env* gene (such as *McClintock* and *accord*) was restricted to nurse cells and the developing oocyte. In contrast, all studied infectious retroviruses were exclusively expressed in the somatic cells surrounding the germline. Intriguingly, endogenous retroviruses displayed distinct, highly spatial and temporal expression patterns within the somatic cell population. For example, *idefix* was restricted to terminal filament cells and cap cells within the germarium, a structure at the anterior of each ovariole where oogenesis starts, whereas *springer* was expressed in follicle cells only of mid-stage follicles. In contrast, *ZAM* displayed a much broader expression pattern in a range of different somatic cell types throughout oogenesis. Interestingly, in the absence of a somatic piRNA pathway, RNA from endogenous retroviruses was also detected in the developing oocyte and, eventually, at the posterior pole of embryos (where integration into the host genome of the progeny likely occurs (Varoqui et al, 2024)), indicating that endogenous retroviruses invade the germline from the surrounding somatic cells.

The authors therefore propose that endogenous retroviruses populate distinct cellular niches within the different somatic cell types of the ovary (Rust et al, 2020) and from there infect the germline. In contrast, loss of *env* correlates with a germline-restricted expression for retrotransposons, allowing conveyance of their genetic information without the need to produce virus-like particles. The authors further suggest that the specific somatic expression patterns are the outcome of niche partitioning, a strategy to avoid competition between different endogenous retroviruses. The exact nature of this competition, however, remains to be investigated. Additionally, whether the concept of niche partitioning also applies more broadly to other families of invertebrate retroviruses (Malik et al, 2000) remains to be explored. Interestingly, a recent study indicated that endogenous retroviruses seem to have specialized not only in their spatiotemporal expression, but also in their preference for different chromatin environments and developmental timepoints for their integration into the host genome (Varoqui et al, 2024), adding to the notion that endogenous retroviruses specialize to occupy highly specific niches. The authors next examined the sequences of *gypsy/gypsy* elements and found the LTRs and 5’ UTRs to be more divergent than the rest of the genome. Both regions harbor *cis*-regulatory sequences that retroviruses and retrotransposons, like cellular genes, require for their own expression. Using reporter transgenes in which the LTR and 5’ UTR of different retroelements were placed upstream of a *LacZ* reporter gene, the authors demonstrated that these two sequences alone were sufficient to recapitulate the spatiotemporal expression of the individual elements. However, the exact nature of these *cis*-regulatory sequences remains elusive. One could speculate that different retroelements adopted binding sites for cell-type specific transcription factors, facilitating their transcription only in a subset of cells. For example, expression of *ZAM* is known to be regulated by the transcription factor Pointed, which indeed is expressed in the same cells (Meignin et al, 2004). Likewise, the endogenous retrovirus *roo* (member of the *Belpaoviridae* family) encodes putative binding sites for multiple developmental transcription factors, and its transcription closely resembles that of these transcription factors during embryonic development (Batut et al, 2013).

Transposons need to be tightly controlled to prevent damage to their host. Due to their differential expression, endogenous retroviruses and retrotransposons encounter different branches of the piRNA pathway. In both germline and soma, large genomic regions containing remnants of transposon integrations, so-called piRNA clusters, produce the majority of piRNAs that silence cognate elements. However, the exact makeup of those clusters differs. In somatic cells, a single uni-strand piRNA cluster of several hundred kilobases in length, known as the *flamenco* locus, is the master suppressor of LTR retroelements. In contrast, in the germline a multitude of clusters transcribed from both genomic strands, called dual-strand clusters, silence a diverse set of transposable elements (Sato and Siomi, 2020) (Fig. 1B). Interestingly, *flamenco*-type piRNA clusters evolved multiple times during drosophilid evolution, and these loci are especially enriched in *env*-encoding endogenous retroviruses (Van Lopik et al, 2023). Senti et al confirmed that *flamenco* is a primary silencer of endogenous retroviruses but not of non-infectious retrotransposons. However, the authors noted that some endogenous retroviruses, such as *17.6* and *idefix*, are either only partially represented or completely absent from *flamenco*. Instead, these elements are silenced by a newly identified uni-strand piRNA cluster, termed *cluster 77B*, which is specifically expressed in a subset of cells in the germarium, the site where the *17.6* and *idefix* elements are active. Strikingly, the mechanism of transcription is different between *cluster 77B* and *flamenco*. While *flamenco* is driven by a canonical promoter under the control of the transcription factors Cubitus interruptus and Traffic jam (Goriaux et al, 2014; Alizada et al, 2025), transcription of *cluster 77B* is driven by the LTR of an old insertion of a *quasimodo* transposable element. This raises the question if additional cell-type-specific piRNA clusters exist in other insects, especially since many species express piRNAs also outside of the gonads (Lewis et al, 2018). Moreover, it will be of interest to study whether repurposing of retroviral elements is a recurring theme for piRNA cluster expression.

An intriguing exception of the dichotomy of endogenous retroviruses and *env*-less retrotransposons is the element *rover*. *Rover* is expressed both in the germline and the soma, and sequences of this element are present in *flamenco*. Close examination of *rover* indicated that most insertions carry a defective *env* gene, yet some insertions still contained an intact *env* open reading frame, and these *rover* variants can be distinguished based on LTR length. Interestingly, the different LTRs together with the 5’ UTRs determined soma and germline expression in a *LacZ* reporter assay. While LTRs of *rover* insertions with intact *env* genes drove expression in somatic cells, the LTRs of *env*-less *rover* elements were exclusively active in germline nurse cells. This suggests that a change in *cis*-regulatory elements in the LTRs underlies the change in expression upon loss of *env* and that *rover* is in a transitional state from an endogenous retrovirus to a retrotransposon.

Together, Senti et al (2025) provide a compelling perspective on the evolution and diversification of endogenous retroviruses and introduce an interesting conceptual framework of the ovary as a complex ecosystem (Fig. 1). The defining, initial capture of an *env* gene from a baculovirus conferred infectiousness to retroelements, allowing them to occupy new cellular niches in the soma and escape germline defenses. The resulting endogenous retroviruses evolved to become active in different somatic cell types in the ovary through adaptive changes in their *cis*-regulatory elements. The more diverse ecosystem of the somatic niche supports a broader retroviral species diversity than the more restricted nature of the germline. Conversely, loss of the *env* gene necessitated adaptation of the regulatory sequences in retroelements to become active again in the germline, where—with time—they became silenced by germline piRNA defenses. This study significantly advances our understanding of the co-evolution of infectious endogenous retroviruses and their hosts. Moreover, the paper and its extensive supplementary figures form a treasure trove of curated information that may be foundational for future work on the principles of endogenous retrovirus evolution and virus-host co-evolution (Alizada et al, 2025).
